# Multiomics Based Association Mapping in Wheat Reveals Genetic Architecture of Quality and Allergenic Related Proteins

**DOI:** 10.3390/ijms24021485

**Published:** 2023-01-12

**Authors:** Khaoula El Hassouni, Muhammad Afzal, Kim A. Steige, Malte Sielaff, Valentina Curella, Manjusha Neerukonda, Stefan Tenzer, Detlef Schuppan, Carl Friedrich Horst Longin, Patrick Thorwarth

**Affiliations:** 1 State Plant Breeding Institute, University of Hohenheim, Fruwirthstr. 21, 70599 Stuttgart, Germany; 2 Institute for Immunology, University Medical Center of the Johannes Gutenberg University Mainz, Langenbeckstr. 1, 55131 Mainz, Germany; 3 Institute of Translational Immunology and Research Center for Immune Therapy, University Medical Center of the Johannes Gutenberg University Mainz, Langenbeckstr. 1, 55131 Mainz, Germany; 4 Division of Gastroenterology, Beth Israel Deaconess Medical Center, Harvard Medical School, 330 Brookline Avenue, Boston, MA 02215, USA

**Keywords:** wheat, proteins, quality, allergen, QTL, breeding

## Abstract

Wheat is an important staple crop since its proteins contribute to human and animal nutrition and are important for its end-use quality. However, wheat proteins can also cause adverse human reactions for a large number of people. We performed a genome wide association study (GWAS) on 114 proteins quantified by LC-MS-based proteomics and expressed in an environmentally stable manner in 148 wheat cultivars with a heritability > 0.6. For 54 proteins, we detected quantitative trait loci (QTL) that exceeded the Bonferroni-corrected significance threshold and explained 17.3–84.5% of the genotypic variance. Proteins in the same family often clustered at a very close chromosomal position or the potential homeolog. Major QTLs were found for four well-known glutenin and gliadin subunits, and the QTL segregation pattern in the protein encoding the high molecular weight glutenin subunit *Dx5* could be confirmed by SDS gel-electrophoresis. For nine potential allergenic proteins, large QTLs could be identified, and their measured allele frequencies open the possibility to select for low protein abundance by markers as long as their relevance for human health has been conclusively demonstrated. A potential allergen was introduced in the beginning of 1980s that may be linked to the cluster of resistance genes introgressed on chromosome 2AS from *Triticum ventricosum*. The reported sequence information for the 54 major QTLs can be used to design efficient markers for future wheat breeding.

## 1. Introduction

Wheat (*Triticum aestivum* ssp. *aestivum*) is the most widely grown crop and a major component of the human diet worldwide. This staple crop is one of the most important sources of energy [1] and on average provides 20% of the total protein and calories in human nutrition [2]. Wheat is consumed in many different forms, and each type of end-product requires a particular quality based on the viscoelastic properties of the dough, which are mainly influenced by the amount and composition of gluten [3]. Gluten accounts for approximately 80% of the total protein in the grain and can be divided into gliadins and glutenins. Glutenins are classified into high and low molecular weight subunits (HMW-GS and LMW-GS) [4], which are encoded by the loci *Glu-1* and *Glu-3*, respectively. Differences between allele pairs in glutenin subunits have a strong influence on the end-use quality [5]. For example, several studies have shown that the alleles *Dx5 + Dy10* (*Glu-D1d*) are associated with high quality, whereas *Dx2 + Dy12* (*Glu-D1a*) lead to poor quality [6,7,8]. Consequently, wheat breeders have intensively selected for specific combinations of HMW-GS since the pioneering work of Payne et al. [9]. In addition to its effect on end-product quality, gluten and non-gluten proteins, such as wheat amylase trypsin inhibitors (ATIs), are also associated with various human health disorders, such as celiac disease, allergic reactions, and non-celiac wheat sensitivity [10,11,12]. However, targeting wheat allergens has never been a goal of breeding programs other than improving gluten quality due to its impact on end-product quality. For instance, the abundance of many allergenic proteins or ATIs in wheat cultivars released in the last century has not changed [13,14]. While only a few proteins have been investigated in detail in recent decades [15], the recent developments in the field of mass spectrometry-based proteomics has led to the possibility to determine hundreds of proteins in a single sample [16]. Afzal et al. [17] analyzed the flour proteome of 15 spelt and wheat cultivars grown in three different locations and identified 3050 proteins, including 300 proteins with moderate-to-high heritability (>0.4). However, to our knowledge, no study investigated the genetic architecture of the large number of proteins that have been discovered with modern proteomic tools so far.

Therefore, we performed a GWAS to investigate the genetic architecture of 114 proteins quantified using liquid chromatography-mass spectrometry (LC-MS)-based label-free quantitative (LFQ) proteomics from 148 bread wheat cultivars grown in three environments. In addition, the genetic and temporal trend of the alleles of some major QTLs associated with relevant proteins were investigated, and the sequence information for relevant QTLs is provided so that it is possible to design molecular markers.

## 2. Results

**Figure 1:**
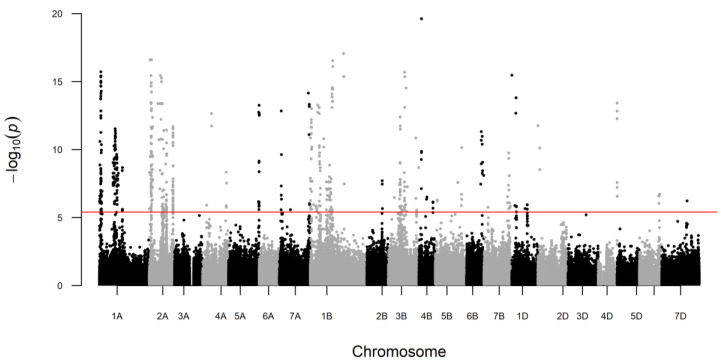
Manhattan plot showing significant marker-trait associations for all 54 proteins, where QTLs at Bonferroni-corrected significance threshold of *p* < 0.05 were identified (red line).

**Figure 2:**
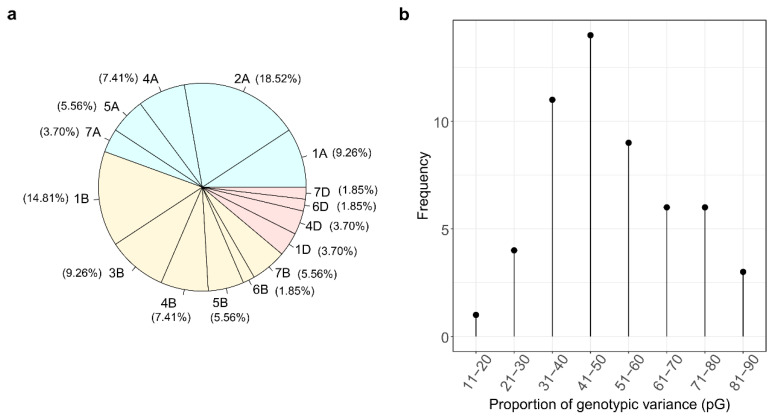
(**a**) Pie chart showing the distribution of the 54 major QTLs on different chromosomes; (**b**) frequency of the proportion of explained genotypic variance by the 54 QTLs.

In a previous study, we measured 756 proteins in aqueous extracts from the whole-grain flour of 148 wheat cultivars grown in three environments [18]. Only 114 of these 756 proteins had a stable expression across environments in most wheat cultivars with a heritability larger than 0.6. For these 114 proteins, we performed a GWAS and detected QTLs for 54 proteins that exceeded the Bonferroni-corrected significance threshold (Figure 1 and Figure 2). For all these 54 proteins, a single major QTL explaining 17.3 to 84.5% of the genotypic variance was identified (Figure 2b, Table 1, Table S1). For 24 proteins, the identified QTLs explained >50% of the genotypic variance (Figure 2b). In contrast, for 60 proteins, no marker-trait association was detected that exceeded the Bonferroni-corrected significance threshold.

**Figure 3:**
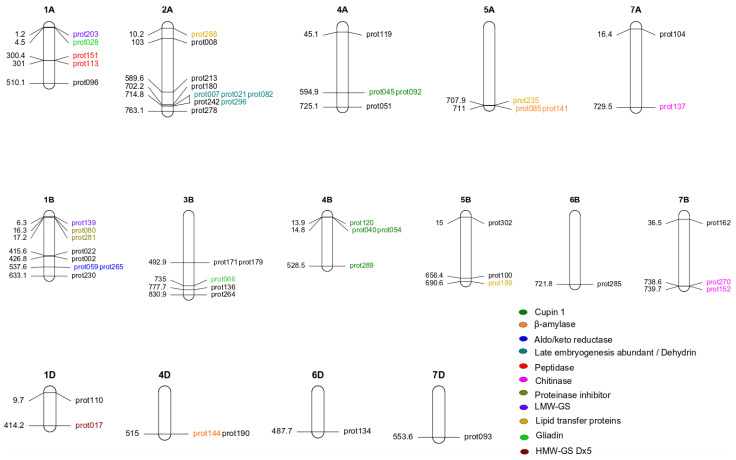
Chromosome map showing the distribution of 54 QTLs with their respective protein name and physical position (Mbp). For a detailed investigation of wheat flour proteins, we focused on the eleven proteins associated with gluten and potential allergenicity based on UniProt and InterPro databases (Table 1). Five of the seven gluten proteins were also present in the Allergome database (https://allergome.org/, accessed on 16 February 2021). We assigned these eleven proteins to the following groups: gluten, gluten and allergen, and non-gluten allergen. Using the UniProt database, we were able to name eight of these eleven proteins, including proteins important for baking quality such as HMW-GS *Dx5* and LMW *Glu-A3*. QTLs for two lipid transfer proteins could be identified, but not for other known, potentially allergenic wheat proteins such as ATIs or serpins.

The major QTLs were distributed across many chromosomes and partly clustered in similar chromosomal regions (Figure 2 and Figure 3). QTLs were identified on chromosomes 1A, 2A, 4A, 5A, 7A, 1B, 3B, 4B, 5B, 6B, 7B, 1D, 4D, 6D, and 7D with a relatively similar distribution on the A and B genomes, whereas only 12% of the identified QTLs were located on the D genome. A higher number of proteins seemed to be affected by the major QTLs on chromosomes 1A, 2A, 1B, and 3B. Interestingly, some QTLs for different proteins were identified at almost the same genomic position (Figure 3, Table S1). For instance, on chromosome 5A, the QTLs for prot085 and prot141, both ß-amylases according to the UniProt database, had the same chromosomal and physical position. Similarly, the QTLs for prot171 and prot179 had the same physical position on chromosome 3B, but according to the various protein annotation databases available, it is not yet clear whether these proteins belong to the same family (Table S1). To design easy-to-use markers for breeding, we have summarized the SNP, genomic position, and sequence information of all identified 54 QTLs in Table S1.

**Figure 4:**
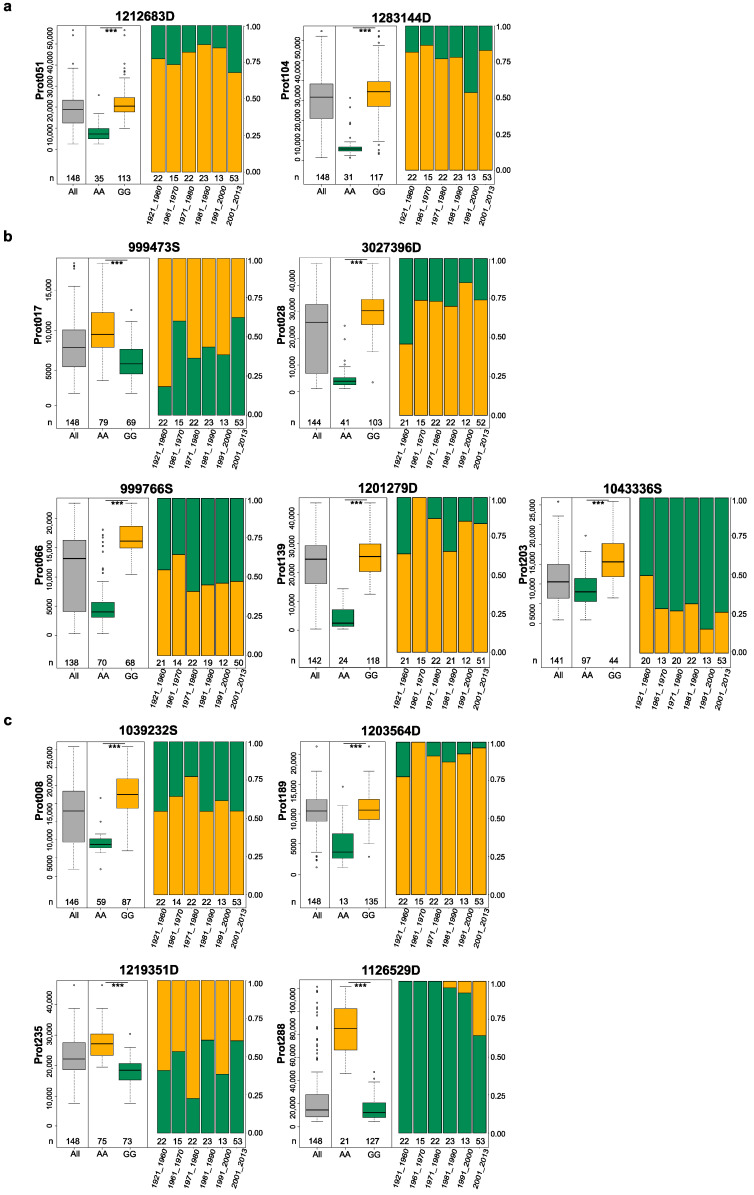
The effect of the major QTLs for gluten proteins prot051 and prot104 (**a**); gluten and allergenic proteins prot017, prot028, prot066, prot139, and prot203 (**b**); and non-gluten allergenic proteins prot008, prot189, prot235, and prot288 (**c**), and their allele frequencies according to the cultivar’s year of release (numbers below boxes represent the number of cultivars in the respective group). The leftmost boxplot in gray show the protein values for all cultivars. The protein-increasing allele is colored in orange; The protein-decreasing allele is colored in green. *** indicates significant difference at *p* < 0.001 between the two groups of cultivars containing contrasting alleles of a given marker.

We further investigated the allele frequencies of QTLs associated with these eleven proteins (Figure 4). For prot008, prot017, prot066, and prot235, allele frequencies of their QTLs were found to be around 0.5. In contrast, for the QTLs of the other seven proteins, allele frequencies tended to a considerably higher frequency of one allele. Interestingly, for five of these seven proteins, the QTLs alleles increasing protein abundance were more frequent. As the wheat cultivars used in this study were released in different decades of the last century, we grouped them accordingly to visualize potential selection trends by wheat breeders. For four proteins, we observed shifts in QTLs allele frequencies across the decades of breeding.

Finally, we investigated the chromosomal regions harboring the QTLs of the 11 proteins in detail (Table S4). For these regions, we extracted high confidence (HC) genes from the bread wheat reference genome (IWGSC RefSeq v2.1) and evaluated these as potential candidate genes with functional annotations in the Pfam and InterPro databases similar to the different domains of gluten and allergenic proteins. Eight, five, and twenty-two potential candidate genes were identified in the QTLs target regions associated with gluten proteins (prot051 and prot104), allergens and gluten (prot017, prot028, prot139 and prot203), and non-gluten allergens (prot189, prot235), respectively (Table S4). No potential candidate genes could be identified for the QTLs detected for the prot066, prot008, and prot288.

## 3. Discussion

### 3.1. Major QTLs Identified for 54 Proteins

Implementing a GWAS using statistically conservative Bonferroni-corrected significance threshold, we identified major QTLs for 54 out of 114 proteins (Figure 1 and Figure 2). Our findings suggest that more than half of the investigated proteins are quantitatively inherited and controlled by many genes, each with rather small effect. This quantitative inheritance is well-described in literature for the most investigated traits, e.g., yield, but also classically determined protein content [19,20,21]. In contrast, many of the QTLs identified for the 54 proteins had very high peaks in the Manhattan plot (Figure 1) and explained a large proportion of the genotypic variance of the individual proteins (Figure 2b, Table 1). In wheat, major QTLs are known, such as for plant height (*Rht* genes), heading time (*Ppd* genes), and disease resistance (e.g., *Lr* genes), but in most cases, the proportion of the explained genotypic variance was much lower than for many proteins in our study [22,23]. Therefore, the identified QTLs could be very interesting for future wheat breeding, provided that the relevance of the respective proteins for future wheat supply chains is demonstrated.

The 54 identified QTLs were similarly distributed across the A and B genomes, but only a small number of them were detected on the D genome (Figure 2a and Figure 3). This is in line with the literature on genomics in wheat [24,25] and can be explained by the limited genetic diversity of the D genome compared to the A and B genomes. Interesting breeding approaches have begun to utilize the genetic potential of the D genome of wheat, such as synthetic wheat [26,27]. As these breeding lines are quite new and, to our knowledge, not yet present in European wheat cultivars, they were also not present in our wheat cultivar list.

For proteins belonging to the same family, we found that they are controlled by loci whose physical positions are located close to one another on the same chromosome or by loci on potential homologous chromosomes (Figure 3). For instance, we identified QTLs for six Cupin 1 proteins, all located on chromosomes 4A and 4B (Figure 3, Table 1). QTLs of proteins 045 and 092 were located on the identical physical map position on 4A, whereas QTLs for proteins 040, 054, and 120 were very close to each other on 4B. We found further QTLs clusters for other protein families. These QTLs are found for three late embryogenesis abundant proteins on 2A, two proteins of the aldo/keto reductase family on 1B, two β-amylases on 5A, and two proteins of chitinase class 1 on 7B. Potentially homologous chromosomal positions were identified for QTLs of two lipid transfer proteins on 5A and 5B, two plant antimicrobial proteins on 6B and 6D, three proteins of chitinase class 1 on 7A and 7B, and for two LMW-GS on 1A and 1B (Figure 3, Table 1). These findings are comparable to other traits where important gene families are located on the same group of homologous chromosomes, e.g., for plant height on chromosomes of group 4 (*Rht1* and *Rht2* genes) or heading time on chromosomes of group 2 (*Ppd-1* genes). In summary, to our knowledge, this largest GWAS study on the wheat proteome revealed a similar genetic architecture of proteins as reported for other traits, with major QTLs for 24 out of 114 proteins.

### 3.2. QTLs for Important Gluten Proteins

High and low molecular weight glutenins are of great importance for wheat end-use quality. They have been under intensive research and use in wheat breeding since the pioneering work of Payne and colleagues [4,9]. We identified three major QTLs underpinning three proteins specifically related to glutenins, one HMW- and two LMW-GS proteins (Table 1). On chromosome 1D, we found a major QTL explaining 42.7% of the genotypic variance for prot017, which is annotated as HMW-*Dx5* according to the Uniprot database (Table 1). Electrophoretic analysis by SDS-PAGE on our wheat cultivars revealed that the QTL allele GG of prot017 was represented by HMW banding unit 5 and the QTL allele AA by the HMW banding unit 2 for a total of 143 out of 148 wheat cultivars (Table S2), confirming the Uniprot annotation. Interestingly, the allelic difference from the SDS banding pattern could be deduced from the quantitative measurements of a single protein (017), where cultivars with the *Dx5* (GG) unit had a lower abundance than cultivars with the *Dx2* (AA) unit (Figure 4, Table S2). While it was not possible to qualitatively distinguish the highly homologous protein isoforms by the tryptic peptides quantified by our mass-spectrometry-based proteomics workflow, the apparently allele-dependent expression level of the gene product was perfectly captured by the complementary SDS-PAGE approach. This case study highlights that quantitative protein measurements can provide additional relevant information content to purely genetic analyses for breeding studies.

In addition, we observed a temporal trend in selection for the QTL alleles of prot017. Wheat cultivars released after 2001 had a higher frequency of the *Dx5* allele (GG) than the *Dx2* allele (AA) compared with wheat cultivars bred before the year 2000 (Figure 4). Wheat breeders have intensively selected for the *Dx5* unit since the 1980s using SDS gel-electrophoresis, increasingly combined with molecular markers [28,29] such as the codominant markers ‘UMN25’ and ‘UMN26’ developed by Liu et al. [30] and the SNP markers used to discriminate Dx subunits developed and validated by Schwarz et al. [31]. However, >30% of modern wheat cultivars in our panel carry the allele *Dx2* (AA), showing further potential for improving baking quality. Since our panel does not contain any very modern wheat varieties, it is likely that breeders have already further increased the frequency of *Dx5*.

The major QTLs identified for two LMW proteins (prot139, prot203) were in similar regions on chromosome 1A and 1B (Figure 3). According to the UniProt database, prot203 corresponds to *Glu-A3* (Table S1), and we speculate that prot139 might be the homologous *Glu-B3* warranting further confirmation. For prot203 (=*Glu-A3*), a selection trend towards fixation of the QTL allele AA was evident over the different decades of wheat breeding (Figure 4). For prot139, the QTL allele that increases protein abundance appears to be close to fixation. The LMW glutenin subunits are much more difficult to identify than the HMW-GS described above due to their complexity, heterogeneity, and similarity to each other, as well as to some gliadins [32], and thus, have not been directly selected by wheat breeders in the last decades. The visible selection trend could come from indirect selection for these proteins by measuring dough and baking quality. Nevertheless, molecular markers capable of distinguishing sixteen different alleles at *Glu-A3* and *-B3* were recently developed [32], which will somewhat facilitate future selection.

Close by the genomic location of *Glu-A3*, we identified a major QTL explaining 82.4% of the genotypic variance of prot028, which is a γ-gliadin according to the UniProt database (Table 1). It is known that *Glu-A3*, *Glu-B3,* and *Glu-D3* are tightly linked to *Gli-A1*, *Gli-B1,* and *Gli-D1*, respectively, the latter representing multigene families encoding γ- and ω-gliadin subunits [33]. As with *Glu-A3*, a selection trend in wheat cultivars towards fixation of the protein-increasing QTL allele was evident for prot028 (Figure 4). Consequently, the selection for dough and baking quality in wheat has continuously modified the frequency of protein abundance of Glu-A3 but also of γ-gliadin. In summary, new proteomic approaches were used to confirm the results of single protein analyses. Those approaches are now able to deliver thousands of proteins per sample [34], paving the way for much deeper exploration of their expression and relationship to agronomic and quality traits in crops.

### 3.3. Possibility to Breed for Low Allergen Content

Although wheat is an important and mostly healthy staple crop, a sizeable number of people suffer from wheat sensitivities, with most potential triggers being proteins [34]. We followed the approach of Zimmermann et al. [35] and Afzal et al. [34] and compiled a list of allergens based on data on seed-borne wheat allergens [36] and the Allergome database (http://www.allergome.org/index.php accessed on 16 February 2021) [37]. For nine proteins from this list, we detected major QTLs in our study (Table 1), which explained between 32.3% and 84.5% of the genotypic variance of the respective protein. Five of these were gluten proteins, two probable lipid transfer proteins, one peroxiredoxin, and one a potential protease inhibitor (Table S1). For three out of these nine proteins, we could observe a selection trend at the major QTLs in the wheat cultivars from the past decades (Figure 4). The marker allele producing high protein abundance was increased for prot288, a protease inhibitor, whereas the marker allele responsible for low protein abundance was increased for prot017, HMW-*Dx5*, and prot203, LMW-GS *Glu-A3*. The latter two are important wheat-quality proteins that plant breeders have intensively selected for, as discussed earlier, and at the same time, appear to be potential allergens for a small number of people. Interestingly, the better baking quality at these two loci appears to be correlated with lower protein abundance, i.e., lower allergen levels.

The selection trend for prot288 is interesting in that the QTL allele, which increases protein abundance was introgressed in the early 1980s and its frequency then steadily increased by wheat breeders. Selection for or against potentially allergenic proteins has never been a goal in wheat breeding. Therefore, this selection trend may be due to the linkage with another target trait in wheat breeding that has been used since the 1980s and is largely influenced by the genomic region on the short arm of chromosome 2A. This chromosomal region contains an introgression from *Triticum ventricosum* that has a roughly comparable history [38]. This introgression carries several important disease-resistance genes (e.g., *Lr37* and *Sr38-Yr17-Lr34*) to various important rust diseases [39,40]. In addition, the introgression also appears to improve yield stability [41] and resistance to rice blast [42], all traits that are of great importance for many wheat breeding programs worldwide. For a large proportion of our wheat cultivars, molecular marker information for the disease resistance cluster *Sr38-Yr17-Lr34* (Table S3) is available, which matches almost perfectly with the different QTL alleles of prot288. Consequently, the increase in the QTL allele that increases the abundance of the potential allergen prot288 could be due to indirect selection of disease-resistance genes nearby. According to the physical positions, our identified QTL is 6 Mbp away from the locus reported for the disease resistance cluster. Future studies will have to show whether this potential linkage can be broken by targeted selection using markers for both loci.

For the QTLs of six potential allergenic proteins, we did not detect clear selection trends over the decades of wheat breeding, but either an almost fixation on the QTL allele causing high protein abundance (prot139, prot189) or similar frequencies of both alleles. This is confirmed by our companion study in which we quantified the absolute protein amounts of eight ATIs by isotopically labeled standard peptides [14]. Therein, major QTLs were identified for monomeric and dimeric ATIs with similar allele frequencies for the monomeric ATI 0.28 but near fixation of the QTL allele responsible for high protein abundance of the dimeric ATI 0.19-like. Consequently, the reported sequence information for the major QTLs identified in both studies could largely facilitate breeding for the low protein abundance of eleven potentially allergenic proteins. Further studies are, therefore, urgently needed to work out the relevance of reducing these proteins abundance for human and animal health, so that the laborious breeding progress for these additional traits can finally be addressed.

## 4. Materials and Methods

### 4.1. Plant Material and Field Experiments

Details of the plant material and field experiments were originally reported by Rapp et al. [43] Briefly, a panel of 148 bread wheat cultivars originating from different European countries and registered between 1921 and 2013 were grown in three different locations in Germany (Hohenheim, Oberer Lindenhof, and Eckartsweier). Field trials were executed using a partially replicated (P-rep) design with a net plot size of 1.25 m^2^. The list of the cultivars and their details are provided in Table S5.

### 4.2. LFQ Proteomic

LFQ bottom-up proteomics data were retrieved from a previous study by Afzal et al. [18], publicly available as ProteomeXchange [44] dataset with the identifier PXD023654, in which the proteomics workflow was described in detail. Briefly, water/salt-soluble proteins were extracted from the full-kernel flours of the wheat samples described in Section 3.1 using a buffer composed of 10 mM sodium bicarbonate and 500 mM sodium chloride dissolved in water (pH 7.8). After centrifugation, 5 µL of the clear supernatant was diluted in 50 mM ammonium bicarbonate and 0.1% (*w*/*v*) RapiGest surfactant (Waters Corporation, Milford, MA, USA) and incubated at 80 °C for 15 min. Proteins were reduced with dithiothreitol (DTT), alkylated with iodoacetamide (IAA) and digested overnight by the addition of trypsin at 37 °C. Samples were acidified with trifluoroacetic acid and desalted using Sep-Pak tC_18_ cartridges (Waters Corporation). Purified peptides were lyophilized and reconstituted in water with 0.1% (*v*/*v*) formic acid (FA). Consecutive LC-MS measurements were performed using a nanoACQUITY ultra-performance LC system (Waters corporation) coupled to a SYNAPT G2-S mass spectrometer (Waters Corporation). Peptides were loaded onto a reversed-phase column (HSS T3 300 µm × 100 mm, 1.8 µm, Waters Corporation, column temperature 55 °C) and separated at a flow rate of 8 µL/min over 15 min using a gradient of 1–36% solvent B, which was acetonitrile with 0.1% (*v*/*v*) FA, while solvent A was water with 0.1% (*v*/*v*) FA. Dimethyl sulfoxide was added to the mobile phase after the column, as previously described [45]. Mass spectra were acquired in data-independent mode by MS^E^ [46]. Raw data were processed using ProteinLynx Global Server v.2.0.3 (Waters Corporation) and searched against a *T. aestivum* protein sequence database (UniProtKB release 2019_02, taxon ID: 4565, 142,700 entries). Postprocessing and LFQ was performed using ISOQuant v1.8 [47], applying a false-discovery rate cut-off of 0.01 at the peptide and protein level and Top3-based [48] protein quantification.

### 4.3. Statistical Analysis of Phenotypic Data

Details of the phenotypic data analysis were described in our previous study by Afzal et al. [18]. A total of 756 proteins were detected across the 148 wheat cultivars grown in three environments. However, only 114 proteins had a stable expression across all environments in at least one cultivar and met the following quality criteria: a heritability > 0.6, missing data < 20%, and expression in >50% and >80% of the cultivars in three and two environments, respectively.

Best Linear Unbiased Estimates (BLUEs) used in our GWAS analyses were estimated across the three environments (locations) assuming fixed genotypic effect in a mixed linear model. All phenotypic analyses were conducted using the statistical software R 3.3.2 [49] and software package ASReml-R 3.0 [50].

### 4.4. Genomic Data and Genotyping

Details of the genotyping approach were originally described by Rapp et al. [43]. Briefly, the cultivars were genotyped using the Diversity Arrays Technology (DArT) genotyping-by-sequencing platform (DArTseq). Two types of markers were delivered by DArTseq, codominant SNP (S) and dominant DArT (D) markers. SNP and DArT markers with more than 25% missing data and a minor allele frequency below 5% were omitted. The remaining missing values were imputed using the package LinkImpute [51]. A selection of 12,203 high-quality markers with a known genetic map positions and common chromosomal position between the wheat reference genome IWGSC RefSeq v1.0 and v2.1 were obtained. These polymorphic DArTseq markers were used for the subsequent genetic analyses. The physical map positions of the markers were retrieved from the Chinese Spring wheat genome (IWGSC CS RefSeq v2.1) implementing BLAST search by using NCBI database keeping the default parameter settings (https://www.ncbi.nlm.nih.gov/assembly/GCF_018294505.1, accessed on 15 February 2022). The default stringent Megablast algorithm did not produce any hits for some markers. We used BlastN algorithm for such markers, which allows a word-size down to seven bases. Implementing BLAST search using any of the two algorithms, when multiple hits for a given marker sequence were returned, only the top hit was kept because of its lowest e-value. In case of ties for the first position between two markers, the top hit was retrieved. In addition to using BLAST to determine positions of the DArTseq sequences, the sequences were mapped against the v2.1 IWGSC assembly [52]. DartSeq reads were first converted to fastq format using custom Python scripts (v.3.9.12) [53]. Reads were then mapped to v2.1 IWGSC assembly [52] using bwa mem v 0.7.17 [54] with the default parameters and the -M flag. Samtools view v1.14 [55] was used to obtain mapped reads with quality > 30 (-q30) and primary alignments (-F256). Bam files were then sorted with samtools sort v1.14 [55] and transformed to bed format using bedtools bamtobed v2.29.2 [56]. Finally, mapped regions were compared to BLAST regions using bedtools intersect v2.29.2 [56].

### 4.5. Genome-Wide Association Study (GWAS)

Genome-wide association analysis was performed with the R package GAPIT (http://zzla.net/GAPIT, accessed on 19 June 2022) [57] using a mixed linear model (MLM) accounting for population structure (Q) through a principal component (PC) analysis and for relationships between individuals through a kinship (K) matrix. Stringent selection of marker-trait associations (MTAs) was performed using the Bonferroni correction (with *p*-value cut-off at 0.05) to avoid false positives. The R package ‘qqman’ [58] was used to create the Manhattan plot representing all the MTAs. The genotypic variance explained by each quantitative trait locus (QTL) was calculated using a linear model fitting the significant MTAs ordered based on the strength of their association. The explained genotypic variance (pG) was calculated as: pG = $\frac{R^{2}adj}{H^{2}}$ [59,60], where, $R^{2}adj$ is the adjusted R^2^ from the linear model, and H^2^ is the heritability of the trait. Only the most significantly associated markers were declared as putative QTLs and reported in the manuscript.

Box-and-whisker plots and barplots were generated using the R package ‘ggplot2’ [61] to examine the effect and the allele frequencies of QTL, respectively. We tested for significant differences between the groups of alleles using the *t*-test with ‘stats’ R package [49]. For the candidate gene search, the latest wheat reference genome (IWGSC RefSeq v2.1) and gene functional annotation information were downloaded from the URGI database [52]. High confidence (HC) genes located within the identified chromosomal regions of the significant QTLs were extracted. The genes with the functional annotation similar to the different domains of gluten and allergens were selected as potential candidate genes.

### 4.6. Verification of Two Major QTLs

Electrophoretic analysis was performed to identify HMW-GS composition of the experimental wheat cultivars. Crushed seeds of each cultivar were used to run the electrophoresis. HMW-GS were separated by sodium-dodecyl-sulfate polyacrylamide gel electrophoresis (SDS-PAGE), which was carried out according to the protein extraction process of Singh et al. [62]. The HMW-GS were identified using the previously proposed nomenclature by Payne and Lawrence [63].

For marker-assisted selection for disease, a marker linked to the rust resistance gene Sr38-Yr1 was used. From the Illumina 25 K Infinium SNP array (SGS Institut Fresenius, TraitGenetics Section, Gatersleben, Germany), a codominant marker IAAV8501 linked with Sr38-Yr17-Lr37 locus (Paul Gruner pers. comm.) located on chromosome 2A (12,327,389 bp) was tested on more than 50% of the cultivars.

## Figures and Tables

**Table 1:** QTLs controlling two gluten proteins, four non-gluten allergenic proteins and five gluten proteins, which were also listed as allergens in Allergome database; identified in this study (HMW = High molecular weight; LMW = Low molecular weight; LTP = Lipid transfer protein).

	Trait	UniProt ID	UniProt Name	Marker	Chr.	*p*-Value	LOD	Gen Pos. (cM)	Phy Pos. Start (bp)	Phy Pos. Stop (bp)	*P_G_*	α-Effect
**Gluten**	prot051	A0A3B6I2R2		1212683D	4A	4.65 × 10^−9^	8.33	2,310,811	725,135,595	725,135,664	45.53	7006.50
	prot104	A0A3B6RB62		1283144D	7A	1.48 × 10^−13^	12.83	211,140	16,051,783	16,366,772	54.70	13,071.91
**Gluten and allergen**	prot017	G1E6K7	HMW-*Dx5*	999473S	1D	1.18 × 10^−6^	5.93	1,492,346	414,170,821	414,170,890	42.73	−1947.51
	prot028	Q94G97	γ-Gliadin	3027396D	1A	1.93 × 10^−16^	15.72	150,899	4,462,173	4,462,215	82.41	12,546.17
	prot066	D2KFH0	Gliadin/avenin	999766S	3B	2.07 × 10^−16^	15.68	1,607,210	734,966,174	734,966,228	82.01	5516.69
	prot139	I1XB56	LMW-GS	1201279D	1B	9.60 × 10^−14^	13.02	182,700	6,301,587	6,301,656	73.82	10,631.02
	prot203	C3VN75	LMW-*GluA3*	1043336S	1A	2.51 × 10^−9^	8.60	149,830	1,235,911	1,235,980	49.85	−12,552.71
**Non-gluten allergen**	prot008	Q6W8Q2	Peroxiredoxin	1039232S	2A	5.61 × 10^−16^	15.25	1,198,150	98,916,927	102,957,935	75.2	6937.00
	prot189	Q2PCC3	LTP	1203564D	5B	7.33 × 10^−11^	10.13	2,583,420	690,570,279	690,570,348	32.32	3056.54
	prot235	Q2PCC5	LTP	1219351D	5A	5.60 × 10^−14^	13.25	2,978,100	707,857,285	707,857,336	56.53	−47,278.73
	prot288	A0A1D5UB33		1126529D	2A	2.51 × 10^−17^	16.60	80,770	5,395,135	10,230,260	84.51	−34,444.07

Chr. Chromosome, Gen Pos. chromosome position in cM, Phy Pos. start/stop sequence position in base pairs according to the bread wheat reference genome (IWGSC RefSeq v2.1), *P*_G_ proportion of genotypic variance explained by the QTL in percent, and allele substitution α-effect.

## Data Availability

The mass spectrometry proteomics data are available via ProteomeXchange with identifier PXD023654.

## Supplementary Materials

The following supporting information can be downloaded at: https://www.mdpi.com/article/10.3390/ijms24021485/s1.
